# Rethinking peer review in medicine: From trust to transformation

**DOI:** 10.2478/jccm-2025-0035

**Published:** 2025-07-31

**Authors:** Dan Longrois, Sacha Rozencwaig, Pierre-Grégoire Guinot

**Affiliations:** Departments of Anesthesiology and Intensive Care, Bichat-Claude Bernard and Louis Mourier Hospitals, Assistance Publique–Hôpitaux de Paris, University Paris Cité, INSERM 1148, Paris, France; Anesthesiology and Perioperative Department, Marie Lannelongue Hospital, Le Plessis-Robinson, Paris Saclay University, France; Department of Anesthesiology and Intensive Care, Dijon University Hospital, University of Burgundy, INSERM UMR1231, CTM Lipness Team, Dijon, France

At the heart of biomedical publishing integrity lies peer review—long regarded as the “gold standard” of scientific validation. Yet, despite its foundational role, peer review today reveals critical shortcomings: inconsistency, lack of transparency, slow turnaround, and susceptibility to bias. As the scientific landscape evolves with rising submission volumes, data complexity, and urgency for rapid knowledge dissemination, it is no longer enough to refine peer review; it must be reimagined.

As editors and long-time participants in academic publishing, we have consistently faced the challenges of writing, reviewing, and managing peer evaluations. Identifying qualified reviewers and synthesizing their feedback into fair editorial decisions remains a formidable task. This editorial outlines our concerns and envisions how artificial intelligence (AI) can enhance—not replace—peer review in medicine. Over 90% of the initial text was generated with ChatGPT Scholar using structured prompts; it has since been extensively revised by the authors.

## A Brief history of peer review: from gentlemen’s clubs to global science

While peer review is now synonymous with scholarly rigor, its formal adoption is relatively recent. Though the Philosophical Transactions (1665) by the Royal Society marks the birth of academic journals, early decisions were often editorial or made by elite gatekeepers. With the post–World War II research boom, funding bodies institutionalized peer review, prompting journals to follow. By the 1990s, it was nearly universal in medical publishing. Yet, the system has remained largely analog in an increasingly digital age.

## Systemic Flaws in Medical Peer Review

Today’s peer review is noble in intent but deeply flawed:

- Bias – Reviewers may favor results aligned with their views (confirmation bias) or favor elite institutions (prestige bias). While anonymizing author identities helps, increasing the number of reviewers provides better safeguards.
- Inconsistency – Divergent reviewer opinions are common, with few standards for review quality. Many reviews are inadequate due to time constraints and lack of reviewer training.
- Delays – Lengthy review cycles hinder the timely dissemination of findings, especially during public health crises.
- Opacity – Anonymous and unaccountable reviews can lead to unjustified rejections or flawed acceptances. Initiatives like publishing review content or reviewer names offer greater accountability and are encouraged.
- Methodological Gaps – Many reviewers lack expertise in statistics or study design, resulting in overlooked errors.
- Publication Bias – Null or negative findings are underreported. Reviewers should verify that registered protocols align with study outcomes.
- Fraud Detection Limitations – Peer review rarely uncovers data fabrication or image manipulation.
- Underuse of Reporting Standards – Guidelines like CONSORT, PRISMA, and STROBE are inconsistently applied.
- Inequity – Reviewer pools often lack diversity, limiting perspectives from women, LMIC researchers, and early-career scientists.
- Reviewer Fatigue – Overburdened reviewers may provide rushed or inattentive feedback.

Such issues are untenable in an era of AI, open science, and increased scrutiny.

## The future is hybrid: AI as co-reviewer, not replacement

By 2025, we are already seeing the emergence of AI-assisted peer review. Tools like StatReviewer (statistical validation; https://www.statreviewer.com), Scite (claim tracing; https://scite.ai), and Proofig (image integrity; https://www.proofig.com) are making reviews faster, more consistent, and more rigorous.

Looking ahead to 2030, we envision a hybrid review system where AI and human expertise work in synergy, each complementing the other’s strengths (4), in a fully transparent and auditable process.

The Table summarizes the possible contributions of AI tools to the process of peer review and suggests the probable roles of the human-in-the-loop.

**Table.** Proposals for the respective roles of AI-enhanced and human peer review

**Function**	**AI Role**	**Human-in-the-Loop Role**
Screening of submitted manuscripts	Triage based on topic, novelty, ethics	Override AI errors; ensure contextual fit
Review assignment	Suggest reviewers based on topic, profiles	Ensure diversity, avoid COI
Statistics	Analyze models, p-values, power	Assess logic, applicability, overfitting
Compliance with the instructions to authors	Thoroughly analyze non-compliance with the instructions and correct	Confirm corrections
Reporting Standards	Check for EQUATOR guidelines compliance	Evaluate nuance and rationale for deviations
Fraud detection	Flag duplicate images, data anomalies	Confirm accuracy, avoid false accusations
Language/Clarity	Improve readability and grammar	Preserve scientific meaning and intent
Verification of the references	Check that references are pertinent and error-free	Override AI in nuanced citation issues
Scientific interpretation	Accelerated summaries from recent publications	Correct AI-proposed syntheses
Final decision	Offer structured recommendation	Formulate accountable accept/reject/revise decisions

AI will not—and should not—replace peer reviewers. But AI may outperform human reviewers for “vigilance” tasks such as compliance with the instructions to authors, methodology, and statistics. The main domains where humans may keep control would be scientific interpretation and the final decision of accepting or rejecting the manuscript.

## Human-in-the-Loop: why expertise still matters

The essence of peer review is judgment—a blend of experience, context, ethics, and clinical insight (5). Only humans can (for the moment, but the authors of this editorial anticipate that even these roles can be assumed by AI tools):

- Interpret whether results are clinically meaningful, not just statistically significant.
- Identify ethical red flags that AI may miss.
- Judge whether a flawed but innovative paper deserves revision, not rejection.
- Hold accountability for editorial decisions—essential in medicine.

Also, keeping human-in-the-loop is essential to training. We would not want the reviewers to overrely on AI for the *hard work* and risk diminishing their capacity to judge or innovate. In this future, human reviewers act as editors, ethicists, mentors, and stewards of trust. They use AI not as a crutch but as an amplifier of insight.

## Rebuilding peer review: from critique to collaboration

To ensure AI-enhanced peer review remains equitable, transparent, and effective, we propose:

- Structured review templates – Adopt universal, checklist-based templates aligned with study types (e.g., randomized controlled trials, cohort).
- AI transparency statements – Journals should disclose how AI was used in manuscript triage or review.
- Reviewer education – Mandatory training in both review principles and ethical AI use.
- Recognition and incentives – Public acknowledgment of reviewers’ contributions through ORCID, Publons, or co-review credits.
- Global inclusion – Actively recruit reviewers from underrepresented regions and career stages to diversify perspectives.
- Post-publication AI feedback – Use AI to scan and flag post-publication comments, errata, or retraction risks.

## Conclusion: the new paradigm of peer review is a transition from gatekeeping to guiding

Peer review in 2030 must shift from a gatekeeping role to a guiding mission—helping authors improve their work, enhancing reproducibility, and accelerating the ethical use of knowledge in patient care. Artificial intelligence will not solve peer review’s problems on its own. But when integrated carefully, with humans at the helm, it can transform a centuries-old process into something faster, fairer, and more fit for the complexity of modern medicine. The future of peer review is not post-human. It is post-frictional—where machines manage mechanics, and humans reclaim the space for judgment, empathy, and trust.

The integration of AI into medical peer review represents an essential evolutionary step toward addressing systemic inefficiencies while preserving the essential human judgment required for complex scientific evaluation. AI systems can efficiently perform initial manuscript screening, identifying submissions that fail to meet basic methodological standards, contain statistical errors, or lack novelty through comparison with existing literature databases. Advanced AI systems can also facilitate better reviewer matching by analyzing manuscript content, author expertise, and reviewer specializations to optimize the peer review assignment process. However, the most promising applications involve AI serving as an augmentative tool that enhances rather than replaces human judgment, providing reviewers with comprehensive background analyses, identifying relevant literature, and flagging potential conflicts of interest or ethical concerns that might escape human attention. This hybrid approach leverages the computational power of AI while maintaining the critical thinking, contextual understanding, and nuanced judgment that characterize expert human review.
